# Clustering of the Deadliest Diseases among Iranian Men from 1990 to 2016: A Growth Mixture Model Approach

**Published:** 2019-08-28

**Authors:** Nasrin Borumandnia, Hamid Alavi Majd, Naghmeh Khadembashi, Serveh Heidary

**Affiliations:** ^1^Urology and Nephrology Research Center, Shahid Beheshti University of Medical Sciences, Tehran, Iran; ^2^Department of Biostatistics, School of Allied Medical Sciences, Shahid Beheshti University of Medical Sciences, Tehran, Iran; ^3^Department of English Language, School of Allied Medical Sciences, Shahid Beheshti University of Medical Sciences, Tehran, Iran

**Keywords:** Global burden of disease, Disease clustering, Men’s health, Iran

## Abstract

**Background:** Paying attention to men’s health seems quite important for a variety of reasons. We evaluated the change of mortality rates due to various causes in Iranian men over the past decades.

**Study design:** A cross-sectional study.

**Methods:** The mortality rates for deadliest causes of diseases among Iranian men during 1990-2016 were extracted from the Global Burden of Disease (GBD) study. Latent Growth Mixture Models (LGMM) were applied to determine subgroups’ cause of death. In this way, the causes within each group showed similar trends of mortality rates over time.

**Results:** The LGMM clustered causes into 4 classes. Diabetes mellitus, hypertensive heart disease and neurological disorders have had increasing trend. Causes in class 2, including diarrhea, lower respiratory and other common infectious diseases, ischemic heart disease, ischemic stroke, neonatal disorders, and other non-communicable diseases manifested a slow decreasing trend. Most causes were allocated to 3rd class with a slow increase in mortality rates over time. Finally, within the last class, transport injuries and unintentional injuries revealed a decreasing trend.

**Conclusion:** Most factors have rising trend, despite the fact that some have shown a very slight downward trend. Consequently, according to the four distinguished clusters resulting from LGMM, it is essential to provide programs to attain the goal of access to prevention, treatment, and support for high-risk mortality factors.

## Introduction

Men's tendency to inappropriate health behaviors, negligence of the search for required medical services, as well as lack of employment in sporting activities and also hazardous working condition, has led to the incongruous state of the so-called silent crisis of health among men's health^1^. This has become such a vital matter within health communities for various reasons. The life expectancy of men is lower compared to women^2^. This could be due to biological differences such as greater oxidative stress, telomere length difference, chromosome X compensation, superior performance of the immune system in women, and the protective effect of estrogen in the female body against various diseases^3^. Likewise, environmental factors such as riskier jobs for men and biological problems such as hormonal differences endanger men's health^3^. Therefore, the promotion of men's health in a highly specialized and systematic way should be taken into consideration more and more.

The first National Men's Health Policy Document for Iran was designed in 2013 by the Ministry of Health and Medical Education^4^. Men tend to be more reluctant to receive health services due to stereotypical beliefs about men abilities and self-confidence and physical strength^5^. In addition, they sometimes even refrain from long-term treatments^3^. Men generally go to treatment centers for diseases such as baldness, impotence, or injuries, and often go unheeded for more serious illnesses such as cardiovascular problems^3^. In general, diseases seriously threaten men's health include cardiovascular disease, prostate, testicular and colon ailments, various cancers and osteoporosis^6^. Men tend to use more cigarettes than women, and they consume more alcohol, which brings their life to an unhealthy status. Compared with women, low-calorie diet, high blood pressure and high cholesterol, diabetes, physical activity abnormalities, alcohol and smoking are all risk factors for heart disease and cancer, which generally threatens the health of men and women^7,8^.

According to the results of a cohort study conducted on an Iranian population, cardiovascular disease (cumulative incidence of death: 18.1%), motor vehicle accidents (cumulative incidence of death: 14.4%), cancer (cumulative incidence of death: 6.9%) and unintentional injuries respectively (cumulative incidence of death: 3.9%), are the main causes of death among Iranians, which is approximately twice as likely to occur among men compared with women ^9,10^.

The Iranian researches search did not lead to finding an article that speculated the mortality rate of Iranian men and the clustering of their changes over the past decades. Thus, we decided to conduct the present study in order to identify subgroups’ cause of death (COD) with similar trend during the last decades. For statistical analysis, the growth mixture models (GMMs) were used to classify COD into classes according to their mortality rate trend from 1990 to 2016 (within a two-year period).

## Methods

### Database

Data used in this study include mortality rates of all 63 causes, compiled from the Global Burden of Disease (GBD) database^11^. Data include mortality rate due to each of 63 causes, from 1990 to 2016 (the data for the years 2018, is not yet available on the site) for Iranian men. Based on GBD study, the mortality rate is defined (per 100000 persons) due to each cause per year. Hence, the main outcome in the statistical modeling is mortality rate for each cause in Iranian men during the period of study. More details about the GBD study and the data can be found elsewhere ^11^.

### Statistical Method

The growth mixture modeling (GMM) method was used to categorize the various causes of Iranian men’s deaths based on their mortality rate changes during the course of the study, in such a way that factors that have similar trend, like growing or decreasing or any other trend be clusters in subgroups. The GMMs are applicable when samples have various trends of outcome during the time. The GMM approaches are used to determine if subgroups exist within the population that follow similar trends over time. Using classifying samples into some trajectory classes based on outcome trend, GMMs takes into account population heterogeneity^12^. The GMM utilizes the following equations for specifying each of the K latent classes:

$$\begin{array}{l} y_{\text{it}}^{k}=\eta_{\text{i}0}^{k}+\eta_{i1}^{k}\lambda_{t}^{k}+\varepsilon_{\text{i}t}^{k}\\ \eta_{\text{i}O}^{k}=\eta_{00}^{k}+\underset{j}{\sum }\beta_{01j}^{k}X_{j}+\varepsilon_{\text{i}0}^{k}\\ \eta_{\text{i}1}^{k}=\eta_{10}^{k}+\underset{j}{\sum }\beta_{11j}^{k}x_{j^{+}}\varepsilon_{\text{i}1}^{k} \end{array}$$

The interpretation of the coefficients in this symbolic representation is as follow: $\eta_{00}^{k}$ corresponds to the estimated overall mean level of the initial outcome in kth class and the average rate of outcome change over time for kth class is showed by $\eta_{10}^{k}$ coefficient ^12^. Intercepts in each class shows the estimated overall mean level of the initial mortality rates. Moreover, ε and $\lambda_{t}^{k}\;$ denotes the measurement error and factor loadings, respectively, which differ across different latent subpopulations. The term of $\underset{j}{\sum }\beta_{j}^{k}x_{j^{\;}}$ considers incorporating a predictor that influences on trends and β denotes the regression coefficient of x ^13^. Intercepts in each class shows the estimated overall mean level of the initial mortality rates, and the average rate of changing in mortality rate in every 2 years (interval between period times) is equal to the intensity of slope in that class. GMM model is used to identify the subgroups of causes with similar mortality rate trends during the past decades among Iranian men’s population.

Statistical analysis was performed, using M-plus software, version 6.12 (www.statmodel.com).

## Results

**Figure 1:**
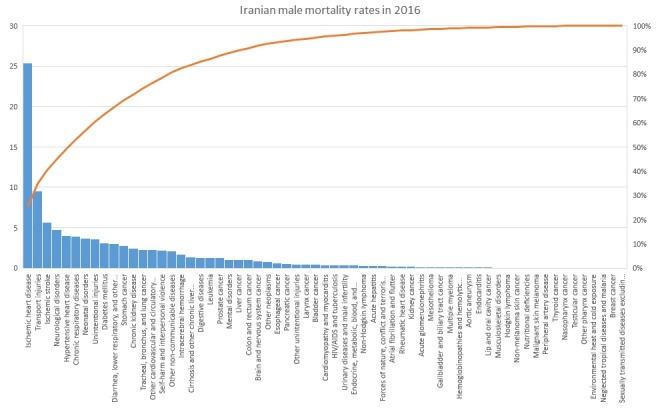
Iranian men’s mortality rate due to various causes in 2016

The mortality rates of various causes for men in 2016 are plotted as Pareto chart in Figure 1. Accordingly, ischemic heart disease, transport injuries and ischemic stroke are the three most commonly reported mortality cases among Iranian men in 2016. Neurological disorders, hypertensive heart disease, chronic respiratory diseases, neonatal disorders, unintentional injuries, diabetes mellitus and diarrhoea, lower respiratory problems, and other common infectious diseases are at the next level. The cause-specific mortality rates are represented in descending order by bars in the chart. The line in graph, showing the cumulative total, reveals that these ten factors account for about 70% of all deaths among Iranian men.

For clustering of COD, GMM was examined through various number of classes, which lead to the following results: clustering with 3 linear classes: AIC=411.77 and entropy=1, clustering with 4 linear classes: AIC=381.39 and entropy=1, clustering with 3 linear classes and 1 non-linear: AIC=102.9 and entropy=1. Finally based on quality of classes and entropy statistics and also AIC, the last model was chosen and the process of model fitting was stopped.

By comparing goodness-of-fit indices for models with different number of classes, model with 4 class was chosen as the best choice. In the chosen model, three classes had linear trend and the other one had non-linear trend (Table 1).

**Table 1:** The results of growth mixture model for clustering of various causes mortality rates of Iranian men

**Class**	**Type of class (Time Scores)**	**Intercept**	**Slop**	***P*** **-value**
1: Increasing trend	Linear: (0,1,2,3,4,5,6,7,8,9,10,11,12,13)	1.52	0.187	0.001
2: Sow decreasing trend	Non-Linear: (0, -40, -92.4, -133.4, -157.9, -177.8, -193.6, -208.1, -213.2, -214.5, -217.1, -224.6, -235.9, -248.7)	10.68	0.011	0.432
3: Slow increasing trend	Linear: (0,1,2,3,4,5,6,7,8,9,10,11,12,13)	0.52	0.010	0.008
4: Decreasing trend	Linear: (0,1,2,3,4,5,6,7,8,9,10,11,12,13)	10.52	-0.200	0.001
Clustering death causes in 4 Classes			
**Class 1:**Diabetes mellitus, Hypertensive heart disease, Neurological disorders			
**Class 2:**Diarrhea lower respiratory and other common infectious diseases, Ischemic heart disease, Ischemic stroke, Neonatal disorders, Other non-communicable diseases			
**Class 3:**			
1. Acute glomerulonephritis	14. Endocarditis, Endocrine, metabolic, blood, and immune disorders	27. Liver cancer	40.Other pharynx cancer
2. Acute hepatitis	15. Environmental heat and cold exposure	28. Malignant skin melanoma	41. Other unintentional injuries
3. Aortic aneurysm	16. Esophageal cancer	29. Mental disorders	42. Pancreatic cancer
4. Atrial fibrillation and flutter	17. Forces of nature, conflict and terrorism, and executions and police conflict	30. Mesothelioma	43. Peripheral artery disease
5. Bladder cancer	18. Gallbladder and biliary tract cancer	31. Multiple myeloma	44. Prostate cancer
6. Brain and nervous system cancer	19. Hemoglobinopathies and hemolytic anemias	32. Musculoskeletal disorders	45. Rheumatic heart disease
7. Breast cancer	20. HIV/AIDS and tuberculosis	33. Nasopharynx cancer	46. Self-harm and interpersonal violence
8. Cardiomyopathy and myocarditis	21. Hodgkin lymphoma	34.Neglected tropical diseases and malaria	47. Sexually transmitted diseases excluding HIV
9. Chronic kidney disease	22. Intracerebral hemorrhage	35. Non-Hodgkin lymphoma	48. Stomach cancer
10. Chronic respiratory diseases	23. Kidney cancer	36. Non-melanoma skin cancer	49. Testicular cancer
11. Cirrhosis and other chronic liver diseases	24. Larynx cancer	37. Nutritional deficiencies	50. Thyroid cancer
12. Colon and rectum cancer	25.Leukemia	38. Other cardiovascular and circulatory diseases	51. Tracheal, bronchus, and lung cancer
13. Digestive diseases	26. Lip and oral cavity cancer	39. Other neoplasms	52. Urinary diseases and male infertility
**Class 4:**Transport injuries, Unintentional injuries			

The second column in Table 1 shows type of each class with its scores. The time scores for the slope growth factor are fixed at 0, 1, 2, up to 13 to define a linear growth model with equidistant time points. Nonlinear class is defined with speciﬁed free time scores to be estimated through the modelling process. The last column shows the coefficients of growth mixture model. Both the estimated intercepts and slopes can help to reveal more about mortality trends among classes. Intercepts in each class show the estimated overall mean level of the initial mortality rates. For example, the estimated overall mean level of the initial mortality rates in class 1 is 1.52%. The average rate of outcome changes in every 2 years (interval between period times is 2 years) for linear class is the intensity of slope in that class.

The results for nonlinear class in Table 1 can be interpreted regarding the speciﬁed free time scores. For example, in class 2, the difference in time scores between 1990 and 1992 is -40, so the change in mortality rates of cancers in this class would be slope*(-40)=(0.011) *(-40)= -0.44, which represents a downward trend of 0.5% during this period of time. In addition, between 2014 and 2016, difference in time scores is -12.8 (-248.7+235.9). Therefore, the change in mortality rates of cancers in this class would be slope*(-12.8) = (0.011) *(-12.8) = -0.14, representing a downward trend of 0.14% from 2014 to 2016. The rest of the results for nonlinear class are interpreted in the same manner.

Class 1 can be defined as having an increasing trend in mortality rates over time. Causes in class 2 experienced a slow decreasing trend of mortality rates over time. Additionally, there was a stable slow increasing trend over time in causes in class 3. Causes in class 4 had an almost sharp trend. The entropy statistics for the clustering has reached 1.00, showing good quality for latent class membership classification.

**Figure 2:**
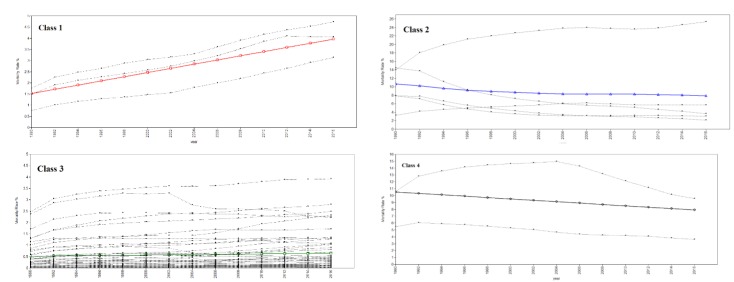
The Iranian men mortality rate trajectories of 63 causes (dashed lines) and the estimated trends from the latent growth mixture model (colored lines)

Growth trajectories for 63 causes have been displayed in Figure 1. Mortality rate trend over time for each cause is shown with a line. As plots show, the trajectories of causes have different trends. According to the GMM results, causes were clustered into 4 classes with different mortality intercepts and trends. The Estimated latent growth trajectories for these 4 classes obtained from GMMs are shown in Figure 2.

## Discussion

It is important to address the issue of Iranian men's health, as negligence on the issue creates considerable and substantial pain and costs; and on the other hand, besides affecting men's quality of life, it has a tremendous impact on life of those around them. Due to the increase in the age of women's life expectancy, they have always witnessed illness and death of their fathers, brothers and sons throughout their lives. In the present study, various COD were clustered based on their mortality trend over past decades among Iranian men.

Based on this study’s results, factors had a large upward trend include diabetes mellitus, hypertensive heart disease and neurological disorders, which require considerable attention. Abdominal obesity is one of the central issues in men's health. Men are more susceptible to obesity than women which causes them to be at risk of serious health problems, including diabetes and hypertensive heart disease ^13^. According to data from adult adolescent clinics of the country's universities during 2015-2016, the proportion of people with type 1 diabetes was 11.4%, type 2 diabetes, 85.5% and other types of diabetes was 1.3%; despite having access to drug and coating insulin use in Iran, the control of this disease and its causes has proved to be difficult^14^. More importantly, the number of people with diabetes mellitus is still increasing for several reasons, such as delay in diagnosis, current treatment failure, genetic factors, lifestyle, and other causes^15^. Cardiovascular diseases directly linked to high blood pressure (both in men and women, particularly in middle age and elderly) are rising^16,17^. Based on the two time periods (2009 and 2015) study on elderly people in Iran, the disease is affected by irreversible risk factors such as age and gender, family history, and other variable risk factors such as obesity, smoking, physical inactivity, hyperlipidemia, and blood pressure ^18^. The burden of disease related to neurological disorders worldwide is increasing, specifically in developing and low-income countries, including Iran^19^. And for reasons such as poverty, lack of awareness, lack of access to low-cost and easy care, lack of accurate knowledge of its epidemiology, and the presence of many people not treated, it surely causes more pressure on society and increase the burden of the disease^20^.

Although based on our results transport injuries and unintentional injuries show a decreasing trend of 0.2%, it has the second-highest mortality rate among other mortality causes. This point has been addressed in another study^21^. Addressing the status of vehicles without safety, the state of insecure roads and finally the timely provision of medical services at the site of an accident are essential here. According to WHO, in 2004, about 3.9 deaths from unwanted accidents occurred worldwide, more than 90% of which are in the middle and low-income countries, with the highest frequency of unwanted incidents. It is related to road accidents, whose control and reduction involves estimating the cost of damage, collecting relevant information, understanding its consequences and engaging with policymakers^22^.

Based on the results of this study’s modeling, factors including diarrhea, lower respiratory problems and other common infectious diseases, ischemic heart disease, ischemic stroke, neonatal disorders and other non-communicable diseases showed a very slow increasing trend of 0.011%, being quite near to zero. Therefore, these factors should be considered and planned for the purpose of reducing their incidence and improving treatment procedures. Diseases such as diarrhea and some other infectious ones categorized in the infectious diseases class have a decreasing and controlled trend due to increased public health and attention to the general health of the community at large, in the advanced and developing countries^23,24^. According to UNICEF, the death rate of infants below the age of five has been dropped from 56 deaths in 1990 to 18 per 1,000 deaths in 2012^25^. The reasons for such result include the development of education and attention to post-natal care and services, promotion of breastfeeding, rehabilitation of newborns, prevention of hyperthermia, and the development of neonatal intensive care units^26^. Currently, cardiovascular disease, followed by cancer and respiratory diseases, as well as stroke as non-communicable diseases are at the forefront of the causes of deaths among men and women worldwide; this can be attributed to lifestyle changes, more industrialization in both developed and developing countries, and the use of ready-made foods, inactivity, smoking and tobacco use^27^.

Finally, based on the results of GMM in this study, the other causes mentioned in class 3 in Table 1, an increasing trend of 0.01% was observed. For some of these factors, such as HIV/AIDS and tuberculosis, the correct number of deaths may not be recorded, which is why there is not much increase expected ^28^. Other studies have shown an increasing trend in this respect. The factors involved in this group may include a small percentage of the total deaths of Iranian men, and yet, they still require attention and planning. Accordingly, it is necessary to provide programs for early detection, screening, preventing, public health program planning, and patient care improvement.

Lack of accurate and reliable registry systems for mortality rate in some of COD in Iran may be considered as a limitation of the present study.

## Conclusion

Despite the fact that more than half of the premature deaths of men can be prevented, there is always evidence of an increase in men's mortality rates, indicating their disregard for preventive factors. Therefore, it is necessary to provide programs to achieve the goal of access to prevention and attention to the factors which have high mortality rates, especially in COD with an increasing trend of mortality rates over time.

## Acknowledgements

The authors sincerely thank the Institute for Health Metrics and Evaluation (IHME) for providing the data.

## Conflict of interest

The authors certify that they have no conflict of interest.

## Funding

The study was funded by Faculty of Allied Sciences, Shahid Beheshti University of Medical Sciences (Grant No. 13260).

## Highlights

- Mortality rate of transport and unintentional injuries showed a similar decreasing trend during 1990-2016.
- Mortality rate of the diabetes mellitus, hypertensive heart and neurological disease had a similar increasing trend.
- Diarrhea lower respiratory, ischemic heart disease, ischemic stroke, neonatal disorders showed a similar slow decreasing trend.
- Mortality rate of other causes revealed a slow increasing trend.
